# Supplementary material for: Phosphorescent extensophores expose elastic nonuniformity in polymer networks
Source: Nat Commun. 2023 Feb 1;14:537. doi: 10.1038/s41467-023-36249-x (PMC9892573; doi:10.1038/s41467-023-36249-x)
Supplement: Supplementary file 1 — Supplementary Information [file 41467_2023_36249_MOESM1_ESM.pdf]

Supplementary information for “Elastic nonuniformity in polymer networks”

Kaikai Zheng,[1] Yifan Zhang,[1] Bo Li,[1] and Steve Granick[1,2*]

[1]Center for Soft and Living Matter, Institute for Basic Science (IBS), Ulsan, South Korea

[2]Department of Chemistry, UNIST, Ulsan, South Korea

This PDF file includes:

Supplementary text describing synthesis of the optical probe.

Figures S1-S4.

Synthesis of the Optical Probe

Anhydrous solvents (chloroform, dichloromethane, DMF, toluene, methanol, ethanol, acetonitrile) were purchased from Daejungchem, China. All other chemicals were purchased from Sigma-Aldrich (2-amino-2-methyl-1-propanol 95%, tert-butyldimethylsilyl chloride 97%, sodium triacetoxyborohydride 97%, sodium bicarbonate 99%, acryloyl chloride 98%, methyl acrylate 98%, benzoin 99%, 1,3-butadiene solution, copper powder, p(THF) Mw=650, 1000) or TCI Chemicals (2,6-pyridinedicarboxaldehyde 98%, 2,6-bis(bromomethyl)pyridine 99%) and used without purification. The scheme is summarized in Figure S1.

Product A: synthesis of TBS protected amino alcohol. Typically, in a flame-dried 100 mL flask under dry argon, alcohol (2-amino-2-methyl-1-propane 3.04 mL, 24 mmol, 1.0 equivalent), tert-butyldimethylsilyl chloride (3.63 g, 24 mmol, 1.0 equivalent) and triethylamine (5 mL, 36 mmol, 1.5 equivalent) are stirred in 100 mL of DCM at room temperature overnight. The next day, 50 mL of sat. $NaHCO_3$ solution are added and the layers are separated. The organic layer is dried over Na_2SO_4, filtered, and concentrated in vacuum. Purification is performed using silica gel chromatography with a gradient eluent from neat DCM to (9:1) DCM/methanol affording desired TBS protection of amino alcohols (*A*, 4.40 g, 90%).

A, 1H NMR (CDCl3, 400 MHz) δ 3.30 (s, 2H), 1.31 (s, 2H), 1.06 (s, 6H), 0.92 (s, 9H), 0.06 (s, 6H); 13C NMR (CDCl3, 125MHz) δ 73.1, 51.2, 26.8, 26.1, 18.5, -5.2.

ESI-MS: (m/z) 203.4.

Product B: synthesis of secondary amine. Typically, a solution dissolving 3.4 g 2,6-pyridinedicarboxaldehyde (25 mmol, 1 equivalent) and products *A*, is prepared by dissolving the 5 g of each reagents (25 mmol, 1 equivalent) in 500 mL of dichloromethane in a 1000 mL flame-

dried flask. The sodium triacetoxyborohydride (10.7 g, 50 mmol, 2 equivalents) is then added to the solution immediately and then stirred overnight. The remaining reactant is destroyed by the addition of 200 mL of aqueous 10% sodium bicarbonate solution. After degasing, the solution is placed into a separatory funnel and the aqueous phase is removed. The organic layer is washed twice more with 100 mL portions of water. Without drying, the solvent is removed by evaporation using a rotary evaporator. This produces products *B* for the next step.

B, 1H NMR (CDCl3, 400 MHz) δ 7.56 (t, 1H), 7.20 (d, 2H), 3.82 (s, 4H), 3.44 (s, 4H), 1.09 (s, 12H), 0.89 (s, 18H), 0.09 (s, 12H); 13C NMR (CDCl3, 125MHz) δ 160.3, 136.8, 119.8, 70.1, 54.0, 48.27, 26.09, 23.91, 18.44, -5.3.

ESI-MS: (m/z) 510.9.

Product C: synthesis of macrocyclic molecule with TBS protection of alcohol. Typically, to a mixture of 2.0 g of the product *B*, (49.16 mmol, 1.15 equivalent) in 10 mL toluene and 10 mL 20% K_2CO_3, solution drops are added as 2.0 g (42.75 mmol, equivalent) of 2,6-bis(bromomethyl)pyridine in 10 mL toluene under 90°C. The reaction (two phases) is performed at 95 °C overnight with viscous stirring. After cooling down to room temperature, the organic fraction is separated and washed with DI water twice. Then, the water phase is removed and the organic phase is moved to the rotary evaporator. The oil-like product is then recrystallized from ethanol and acetonitrile to give the desired products *C*.

Yield: *C*, 1.71g, 85%.

C, 1H NMR (CDCl3, 400 MHz) δ 7.04 (t, 2H), 6.72 (d, 4H), 4.04 (s, 8H), 3.67 (s, 4H), 1.28 (s, 12H), 0.93 (s, 18H), 0.09 (s, 12H); 13C NMR (CDCl3, 125MHz) δ 159, 135.22, 122.00, 69.53, 59.56, 58.09, 26.05, 23.59, 18.40, -5.37.

ESI-MS: (m/z) 613.3.

Product D: removal of TBS protection from alcohols. 2 g of *C* and 5 mL of 2.6 M HCl solution are combined in 30 mL methanol. After stirring the mixture for 30 min, 5 mL of 36% aqueous HCl are added and the reaction is set at 45°C for 10 hr. The mixture is washed with aqueous K_2CO_3 three times and the resulting solution is extracted with dichloromethane (three portions of 100 mL). Drying with anhydrous K_2CO_3 and evaporation of the solvent produces yellow powder *D* (1.9 g 95%).

D, 1H NMR (CDCl3, 400 MHz) δ 7.04 (t, 2H), 6.50 (d, 4H), 3.92 (s, 8H), 3.54 (s, 4H), 1.29 (s, 12H); 13C NMR (CDCl3, 125MHz) δ 159.84, 135.22, 122.00, 69.53, 59.09, 55.69, 22.98.

ESI-MS: (m/z) 385.5.

Product E: synthesis of final product. Typically, to a solution of *D* (1.0 g, 2.57 mmol, 1 equivalent) and triethylamine (2 mL) in dry DCM (20 mL) are added dropwise a dry DCM solution (10 mL) of acryloyl chloride (1.62 ml, 2 mmol, 8 equivalents) at 0 degree Celsius. After stirring at room temperature for 24 h, the solution is poured into water (50 mL) and extracted with diethyl ether (2×30 mL). The combined organic layer is washed with water (50 mL), dried over anhydrous K_2CO_3 and the organic solvent evaporated under reduced pressure. Crude product is purified by flash chromatography on neutral alumina eluting with DCM to give a slight brown oil (1 g, 45%). Then, the product is dissolved in DCM and mixed with Cu(I)I for 5 hr, thus producing the product *E*.

E, 1H NMR (CDCl3, 400 MHz) δ 7.04 (t, 2H), 6.50 (d, 4H), 6.43 (m, 2H), 5.83 (m, 2H), 4.06 (s, 4H), 3.97 (s, 8H), 1.09 (s, 12H); 13C NMR (CDCl3, 125MHz) δ 166.7, 159.9, 135.2, 131.2, 128.2, 122.0, 72.2, 59.1, 52.3, 23.0.

ESI-MS: (m/z) 557.5.

Polymerization. The cross-linked polymethacrylate (PMA) network is produced by free radical addition polymerization of methyl acrylate using benzoin as photo initiator. Methyl acrylate (1 ml), 1μl diluted *E* solution (1 μg/ml in THF), 5 μl diluted1,3-butadiene solution (1.5 mM in toluene), and benzoin (1.75 mg) are well mixed before addition to a Teflon mold (5 cm×1.5 cm×3 mm) with a glass plate covering it. Polymerization takes place by 365 nm UV irradiation (6W lamp) in a glove box for 5 min at room temperature. The low molecular weight substances, unreacted benzoin and crosslinkers remaining in the samples are extracted with THF (100 ml) at room temperature, which is exchanged 24 times during 72 h. The film is then dried under vacuum. Then the film is cut into a rectangular shape using a cutter 6 mm in length and 3 mm in width (w). Finally, it is annealed at 60 ˚C overnight to remove residual stress.

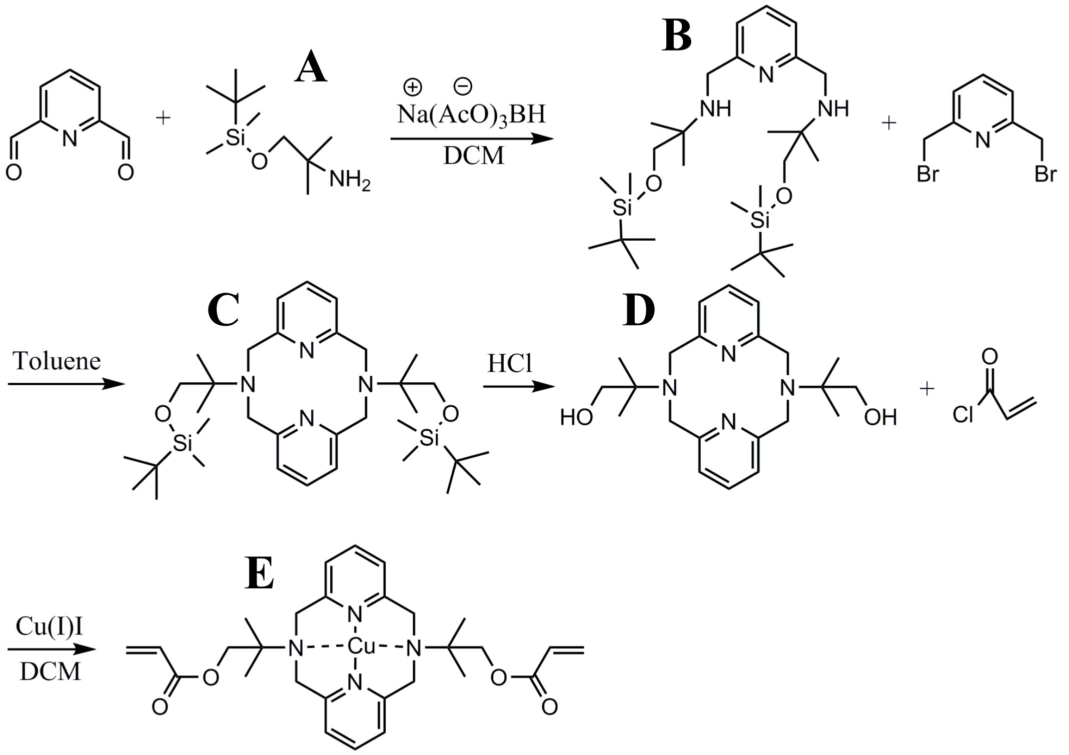

Supplementary Fig 1. Scheme to synthesize the optical probe.

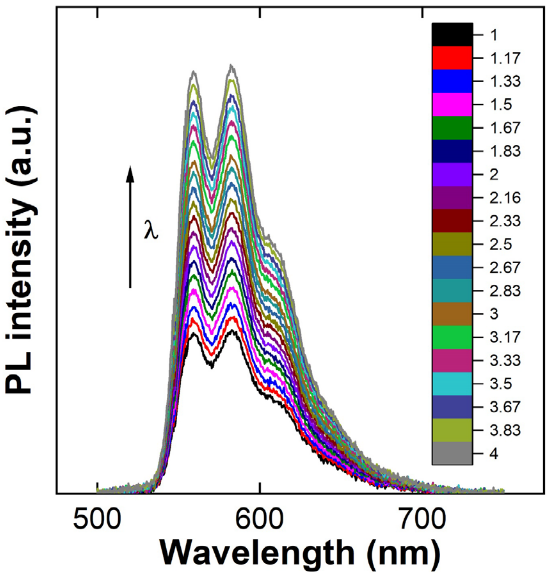

Supplementary Fig 2. Phosphorescence spectrum of a bulk sample at 19 increasing elongation ratios from 1 to 4, color-coded according to the inset.

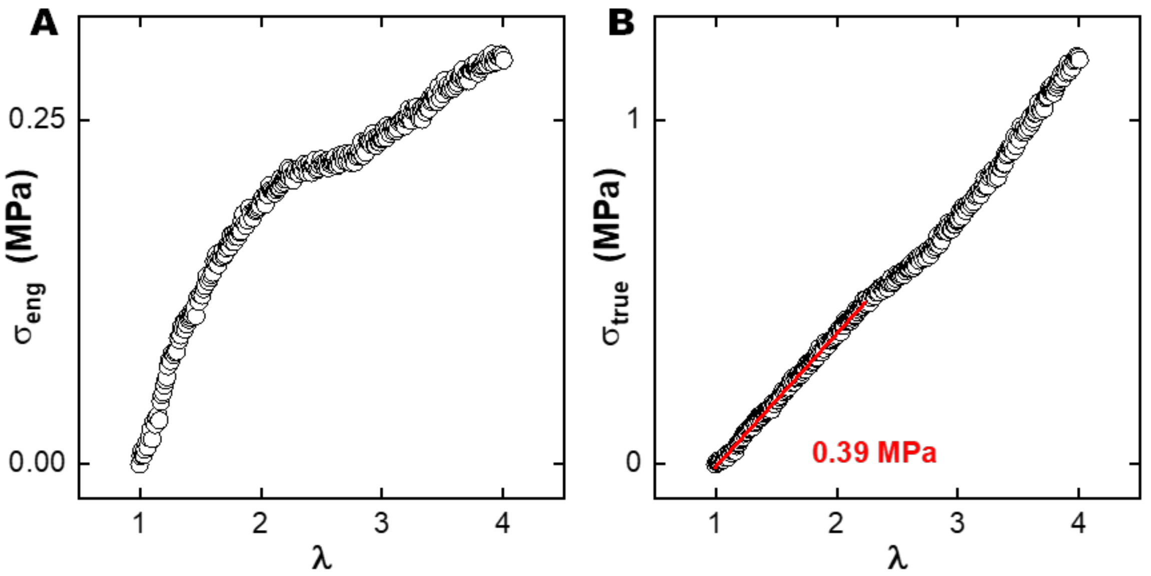

Supplementary Fig 3. Stress-strain curves illustrating the PMA network used in this study. (A) The engineering stress is plotted again strain. (B) For the same data, the true stress is plotted against strain, showing the Young's modulus E obtained from the slope at small extension ratio λ. For rubbers, shear modulus is G = E/3. The speed of stretch from one elongation to the next is 0.5 mm/min, amounting to strain rate 3×10^{-3} s^{-1}, less than the inverse Rouse relaxation of polymer strands between crosslinks.

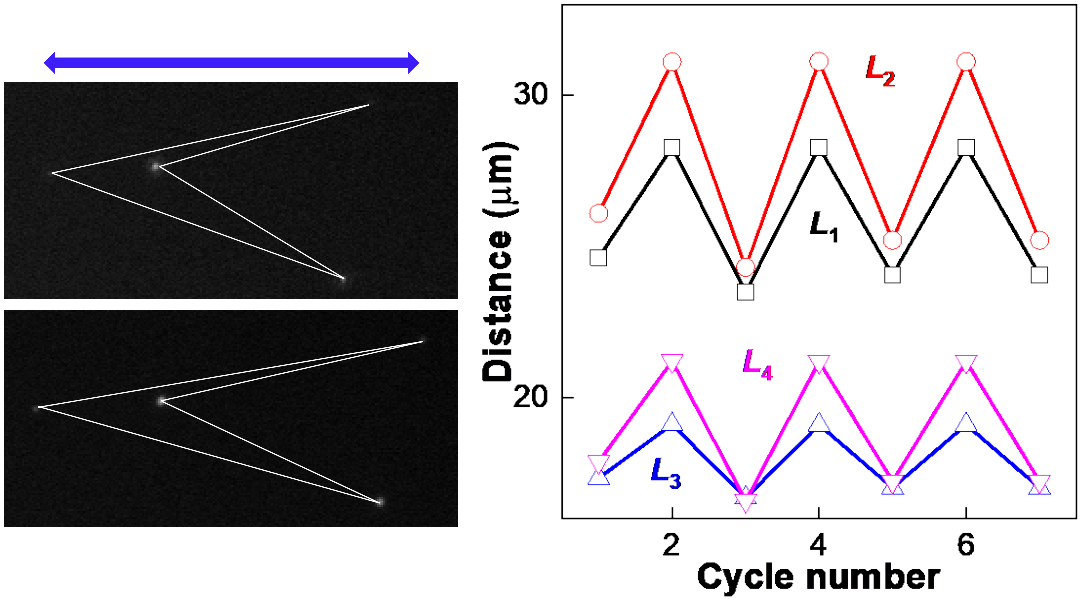

Supplementary Fig 4. Crosslink positions change reversibly during periodic cycles of elongation-contraction. This example with 4 crosslinks maps the distances (*L*) between them.
