# Supplementary material for: Phosphorescent extensophores expose elastic nonuniformity in polymer networks
Source: Nat Commun. 2023 Feb 1;14:537. doi: 10.1038/s41467-023-36249-x (PMC9892573; doi:10.1038/s41467-023-36249-x)
Supplement: Supplementary file 3 — Description of Additional Supplementary Files [file 41467_2023_36249_MOESM3_ESM.pdf]

Supplementary Movie 1. This video of a PMA network prestretched to extension ratio 4 shows five phosphorescent optical probes imaged over 100 seconds.
